# Sex-Dependent Effects on Influenza-Specific Antibody Quantity and Neutralizing Activity following Vaccination of Newborn Non-Human Primates Is Determined by Adjuvants

**DOI:** 10.3390/vaccines12040415

**Published:** 2024-04-15

**Authors:** Beth C. Holbrook, Elene A. Clemens, Martha A. Alexander-Miller

**Affiliations:** Department of Microbiology and Immunology, Wake Forest University School of Medicine, Rm 2E-018 Biotech Place, 575 North Patterson Ave., Winston-Salem, NC 27101, USA; bcholbro@wakehealth.edu (B.C.H.); elene.a.-clemens@uth.tmc.edu (E.A.C.)

**Keywords:** influenza vaccine, adjuvant, newborn, antibody, sex-dependent differences

## Abstract

A number of studies have demonstrated the role of sex in regulating immune responses to vaccination. However, these findings have been limited to adults for both human and animal models. As a result, our understanding of the impact of sex on vaccine responses in the newborn is highly limited. Here, we probe this important question using a newborn non-human primate model. We leveraged our prior analysis of two cohorts of newborns, with one being mother-reared and one nursery-reared. This provided adequate numbers of males and females to interrogate the impact of sex on the response to inactivated influenza vaccines alone or adjuvanted with R848, flagellin, or both. We found that, in contrast to what has been reported in adults, the non-adjuvanted inactivated influenza virus vaccine induced similar levels of virus-specific IgG in male and female newborns. However, the inclusion of R848, either alone or in combination with flagellin, resulted in higher antibody titers in females compared to males. Sex-specific increases in the neutralizing antibody were only observed when both R848 and flagellin were present. These data, generated in the highly translational NHP newborn model, provide novel insights into the role of sex in the immune response of newborns.

## 1. Introduction

An individual’s sex has been shown to have a significant impact on immune responses [1,2]. Females exhibit stronger antiviral responses [3] and higher antibody responses following vaccination [4], including influenza [5,6]. The sex-associated differences in the immune response are attributed to the divergent hormone levels in males and females. Gonadotropins have effects on multiple cells of the immune system, including dendritic cells, B cells, and T cells [7]. For example, estradiol signaling in dendritic cells results in increased TLR-mediated cytokine production [7]. Signaling through the estrogen receptor α in CD4^+^ T cells drives proinflammatory cytokine production and, in B cells, promotes class-switch recombination and somatic hypermutation [7]. In contrast to the often proinflammatory effects of estrogen, testosterone can dampen the immune response. Androgen receptor binding in CD8^+^ T cells represses IFN-γ and granzyme B, and in CD4^+^ T cells, testosterone can inhibit differentiation into Th1, Th2, and Th17 subsets [7].

The level of gonadotropins is relatively low in human newborns during their first week of life [8]. Around 1 week of age, newborns begin to experience a phenomenon termed mini puberty, which results in increases in gonadotropins that peak by 3 months of age [9,10]. This phenomenon also occurs in non-human primate infants [11]. Interestingly, estradiol levels are high in the cord blood of mothers, independent of the sex of the infant. However, levels rapidly decrease in newborns over the first days following delivery [8,12,13] and by 1 week are low in both sexes. Following this nadir, estradiol increases in females [8], and continued increases during the following months result in levels that are higher than those in males [8,14]. Testosterone is minimal in both males and females in the first weeks following birth, after which it increases significantly selectively in males [7]. Thus, shortly following birth, males and females differ in their hormonal profiles.

Sex-dependent alterations have also been reported following the engagement of a subset of TLRs, including TLR4, TLR7, TLR8, and TLR9 [15,16]. The increased sensitivity of TLR7/8 in females is at least in part the result of the higher expression of these TLRs in women due to their escape from X inactivation [17,18], a process that occurs during fetal development to equalize the dosage of gene products between the sexes through the transcriptional silencing of one X chromosome [19].

At present, how these sex-dependent differences manifest in newborns is poorly understood. Recent findings leave open the possibility that the increased sensitivity to TLR ligands that results from the increased expression of TLR7 and TLR8 in females may be mitigated by high levels of miR-146a in newborns. In newborn mice, miR-146a is highly upregulated by 1 day following birth [20]. The expression of miR-146a dampens TLR signaling by inhibiting the expression of IRAK-1, which is a critical component of this pathway [20].

To probe the role of sex in newborn vaccine responses, we assessed the response of newborn non-human primates to vaccination with inactivated influenza virus alone, or adjuvanted with R848 (a TLR7/8 ligand), flagellin (a TLR5 ligand), or a combination of the two. Newborn infants were vaccinated in two cohorts, either nursery-reared or mother-reared. These two cohorts were individually assessed, and we previously reported the effects of these adjuvants on the antibody response [21,22,23,24,25,26]. Although we had both males and females in each cohort, the number of animals in each vaccine group was inadequate to address the impact of sex. However, we could leverage our finding that the responses were similar for each vaccine type in the two cohorts to combine the results and probe the role of sex in the response to each of the vaccines. Here, we report the finding that there were no significant differences between males and females when administered the inactivated virus alone. However, the inclusion of R848 alone or in combination with flagellin resulted in higher amounts of the total influenza-specific IgG antibody in females compared to males. Finally, neutralization titers were only increased in female newborns when both R848 and flagellin were present. Thus, in newborns, females only gave evidence of a superior response when adjuvant was included and not the inactivated virus alone.

## 2. Materials and Methods

### 2.1. Animals

African green monkey (AGM) infants (Caribbean-origin *Chlorocebus aethiops sabaeus*) used in this study were housed at the Vervet Research Colony at Wake Forest School of Medicine. Nursery-reared newborns were removed from their mothers at 1–3 days of age and moved to the nursery. Newborns in the nursery were bottle fed, and care was provided around the clock. Newborns were initially housed in incubators and subsequently moved to a cage when they were capable of thermoregulation. Animal health was assessed by monitoring body weight, temperature, respiration rate, heart rate, food intake, and activity throughout the experiment. Animals were allowed to acclimatize to the nursery prior to receiving the vaccine. Mother-reared newborns remained in the colony in group housing. Infants enrolled in the studies were >300 g and were deemed healthy, as assessed by a veterinarian at check-in following birth.

### 2.2. Animal Approval

All animal protocols were approved by the Institutional Animal Care and Use Committee at Wake Forest University School of Medicine. The WFUSM animal care and use protocol adhered to the U.S. Animal Welfare Act and Regulations.

### 2.3. Influenza A/PR/8/34 (H1N1)

A/Puerto Rico/8/1934 [H1N1] (PR8) virus stocks were grown and titered (as an egg infectious dose (EID_50_)) in fertilized chicken eggs and stored at −80 °C.

### 2.4. Vaccination and Sampling

At 3–6 days of age, infants were vaccinated with 45 μg of the inactivated R848 conjugated PR8 virus (IPR8-R848), IPR8 admixed with 10 μg of flagellin (flg), IPR8 with both adjuvants (IPR8-R848+flg) or IPR8 admixed with 10 μg of inactive 229 mutant flagellin (m229) [27]. Control animals received PBS. The inactivation of PR8 was achieved by treating it with 0.74% formaldehyde overnight at 37 °C. For the IPR8-R848 conjugate vaccine, an amine derivative of R848 (heretofore referred to as R848) was linked to SM(PEG)_4_ by incubation in DMSO for 24 h at 37 °C [23]. R848-SM(PEG)_4_ was then incubated with the influenza virus (PR8-R848). Non-conjugated R848 was removed by dialysis, followed by inactivation with 0.74% formaldehyde as above. Successful conjugation was assessed by the differential stimulation of RAW264.7 cells following incubation with similar amounts of the R848-conjugated versus non-conjugated vaccine. Flagellin from *Salmonella enteritidis* was prepared as previously described [27]. All injections were delivered intramuscularly in the deltoid muscle (500 μL volume). Animals were boosted 21 days later. For sampling, animals were sedated with isoflurane, and blood was collected in sodium heparin tubes by venipuncture on d21 post-prime and boost.

### 2.5. ELISA for the Detection of Influenza Virus-Specific Antibody

Nunc MaxiSorp Elisa plates were coated with 1 µg/well PR8 or 5 ng of a headless A/California/4/2009 (H1N1) HA stabilized stem construct [28]. Plates were blocked with a 1x Blocking Buffer (10x Blocking Buffer, Sigma, St. Louis, MO, USA) plus 2% goat serum (Lampire Biologicals, Pipersville, PA, USA). Plasma samples were serially diluted in a 1x Blocking Buffer. Wells without virus served as a negative control. An HRP-conjugated antibody specific for monkey IgG (Fitzgerald) was used to detect the bound antibody. Plates were developed with 3,3′,5,5′-Tetramethylbenzidine dihydrochloride (Sigma) and read at 450 nm on a BioTek Elx800 Absorbance Microplate Reader. Absorbance for each dilution was calculated by subtracting the OD value obtained for the corresponding non-virus-coated wells. The threshold titer was defined as the value that reached 3 times the assay background, i.e., wells that received only the sample diluent.

### 2.6. Neutralization Assay

Heat-inactivated (56 °C for 1 h) samples were serially diluted in a sterile 96-well flat bottom plate. A total of 7.5 × 10^6^ EID_50_ of PR8-GFP ([29], kindly provided by Dr. Adolfo Garcia-Sastre) was added to each well and incubated for 2 h at 37 °C and 5% CO_2_ to allow for antibody binding. Then, 2 × 10^5^ U937 cells were added to each well and incubated overnight at 37 °C. The next morning, samples were acquired on a BD FACSCalibur and analyzed with CellQuest Pro software Version 6.0 (Becton Dickinson, Franklin Lakes, NJ, USA) to determine the percentage of U937 cells that were positive for GFP. Controls for each experiment consisted of U937 cells alone and U937 cells infected with the PR8-GFP virus in the absence of plasma. The maximal %GFP was calculated for each experiment, and nonlinear regression (GraphPad Prism, Boston, MA, USA) was used to determine the dilution at which 50% maximum PR8-GFP-infected U937 cells were achieved.

### 2.7. Statistical Analysis

Data were analyzed using Prism 5 software (GraphPad). Vaccine groups in the original studies (nursery and mother-reared cohorts) contained 5–8 animals. After the initial studies, we determined that eight animals would provide 80% power to detect a difference between groups of 2.26 titer units for a single time point. This was based on our estimate of the variability in IgG measures. In the earliest studies where the variability was not known, some groups had fewer animals (n = 5–7).

## 3. Results

### 3.1. Sex-Dependent Differences Following the Influenza Vaccination of Newborn NHPs Were Apparent with a TLR7/8 Agonist but Not with a TLR5 Agonist or in the Absence of Adjuvants

Nursery- or mother-reared newborn (3–6 days old) African green monkeys (AGM) were vaccinated with the inactivated PR8 influenza virus (IPR8) adjuvanted with R848 or flagellin. R848 (resiquimod) is a synthetic TLR7 and TLR8 agonist. Salmonella enteritidis-derived flagellin is a TLR5 agonist. Newborn AGM received IPR8 admixed with flagellin (IPR8+flg), IPR8 conjugated with R848 (IPR8-R848) [23], or IPR8 with both adjuvants (IPR8-R848+flg). A group of newborns received IPR8 mixed with an inactive flagellin protein (m229). This mutant flagellin cannot signal through TLR5 [30] and, thus, serves as a non-adjuvanted control. These two cohorts were established to evaluate long-lived antibody responses (mother-reared) or to perform challenge studies (nursery-reared). We found that the influenza-specific IgG responses were similar in animals from the mother-reared and nursery-reared groups (Supplementary Figure S1). This result allowed us to combine the data for each vaccine group, resulting in adequate numbers to analyze them by sex.

**Figure 1:**
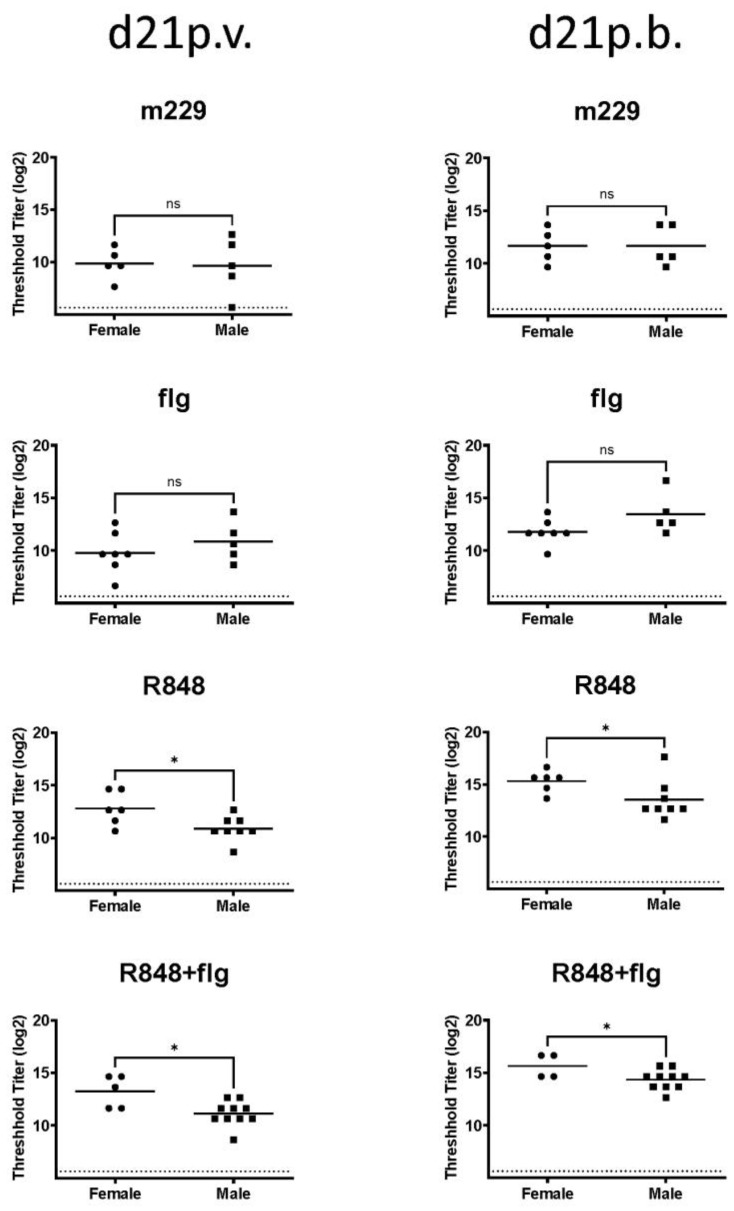
Female newborn AGMs vaccinated with an R848-containing vaccine have increased influenza virus-specific IgG compared to their male counterpart. The 3–6 day-old AGMs were administered IPR8+flg, IPR8-R848, IPR8-R848+flg, or IPR8+m229 (as a non-adjuvanted control). The amount of influenza-specific IgG in plasma was measured at d21 following vaccination and boost. One female animal administered IPR8-R848+flg was removed from the study for unrelated health reasons and, thus, is not included in the d21p.b. data. The value for individual animals (threshold titer = the lowest dilution at which sample OD was at least three times that of the assay background) and the geometric mean of each group are shown. The limit of detection (dotted line) is defined as the lowest sample dilution in the assay. Statistical significance was determined using an unpaired Student’s *t*-test of the log2-transformed values or the Mann–Whitney test where data contained an outlier, as determined by outlier analysis in Prism. * *p* < 0.05, ns = not significant.

The data in Figure 1 show PR8-specific IgG in male and female newborns from each vaccine group at d21 following the initial vaccine dose and d21 following the boost. No difference in the antibody was detected between female and male newborns receiving the non-adjuvanted (m229) or flagellin-adjuvanted vaccine groups. However, in samples from newborns that were administered a vaccine that contained R848 (IPR8-R848 and IPR8-R848+flg), a significantly increased level of the antibody was detected in female animals.

### 3.2. The Sex-Dependent Differences in Anti-Influenza Virus IgG Were Not Observed in the HA Stem-Specific Response 

**Figure 2:**
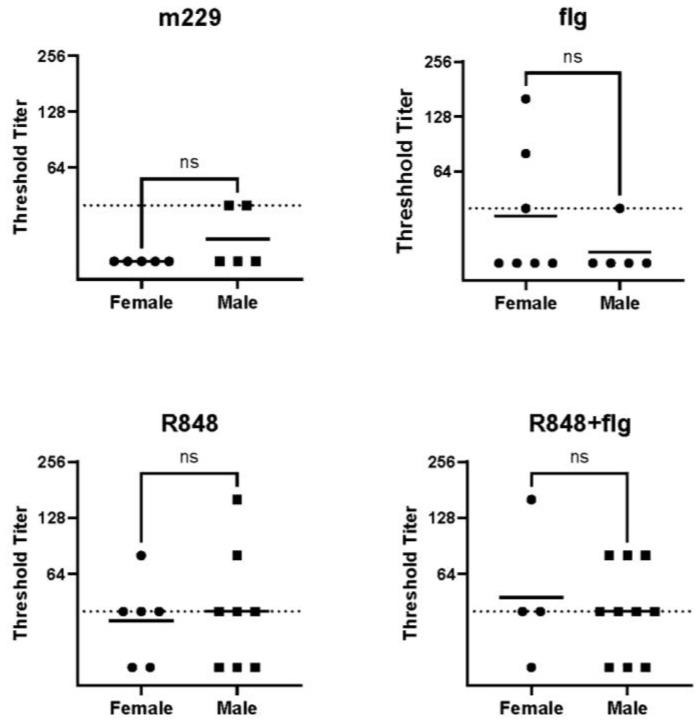
No sex-dependent differences in HA stem-specific IgG are present following vaccination with adjuvanted vaccines. Newborn AGMs were vaccinated and boosted, as shown in Figure 1. Plasma IgG titers to the Ca09 HA stem were measured by ELISA at day 21 p.b. The value for individual animals (threshold titer) and the geometric means are shown. The limit of detection (dotted line) is defined as the lowest sample dilution in the assay. Statistical significance was determined using an unpaired Student’s *t*-test of the log2 transformed values. ns = not significant.

Antibodies to the structurally conserved but immunologically subdominant HA stem are considered a highly beneficial component of the antibody response to influenza virus. This is due to the broadly reactive nature of these antibodies, which confers protection across heterologous strains [31,32]. Given the difference in the virus-specific antibody observed in female newborns receiving vaccines containing R848, we asked whether this was also the case for antibodies to the HA stem region. We quantified this response in the vaccinated male and female newborns using a headless HA stem developed in the laboratory of Dr. Barney Graham [28]. Samples collected 21 days following the boost were assessed as this was the time point at which the highest percentage of animals had a response to this subdominant epitope. In contrast to what was observed with the anti-viral response, no increase in the stem-specific antibody was present in female vs. male newborns (Figure 2).

### 3.3. The Combination of R848 and Flagellin Promotes a Higher Neutralizing Antibody in Females Compared to Male Newborn NHPs 

**Figure 3:**
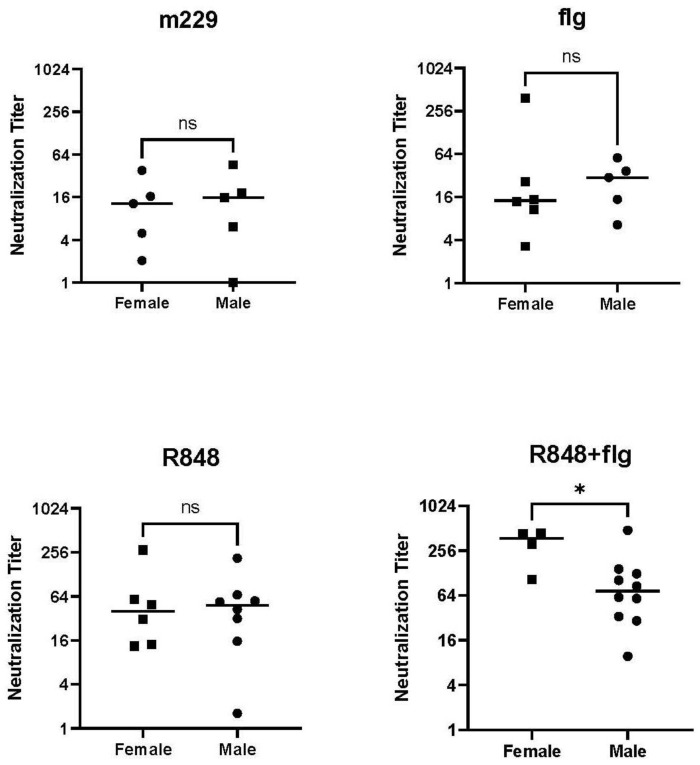
An increased neutralizing antibody in females is only observed when both adjuvants are included in the vaccine. The neutralizing antibody in plasma-obtained d21 p.b. was measured by the inhibition of infection of a GFP-expressing PR8 virus. Titers for individual animals, along with the geometric means, are shown. One female administered IPR8+flg assessed above had an insufficient sample volume, and no sample was available for one IPR8-R848+flg female. Statistical significance was determined using an unpaired Student’s *t*-test. * *p* < 0.05, ns = not significant.

The capacity for antibodies to prevent infection is an important attribute of the ability to control influenza virus infection. Given the critical nature of this antibody function, we evaluated the role of sex in the generation of neutralizing antibodies. We assessed responses at the time point that reflected the longest period for GC responses to generate a high avidity neutralizing antibody, d21 post boost. The data in Figure 3 show that the combination of flagellin and R848 resulted in significantly higher levels of the neutralizing antibody in female newborns. No sex-dependent difference was present with non-adjuvanted, with flagellin-only adjuvanted, or R848-only adjuvanted vaccines.

## 4. Discussion

Newborns and young infants are a major population targeted by vaccination. While information is emerging about the role of sex in vaccine responses in adults, data derived from young infants is highly limited. Using a model of the inactivated influenza virus vaccine administered to newborn NHPs, we provide new insights into this question. We found that in the absence of adjuvants, the antibody response generated following vaccination with inactivated influenza virus was similar in males and females. However, the inclusion of a TLR7/8 agonist resulted in higher influenza-specific IgG in females. This effect was not a general property of TLR agonists, as the addition of flagellin did not stimulate a higher antibody in female animals.

HA, stem-reactive antibodies are of high interest as they have the capacity to provide cross-strain protection. In our analysis of this response, in contrast to what was observed in the whole virus, we found no evidence of sex-dependent effects on the quantity of HA stem-specific IgG generated with any of the vaccine adjuvant combinations. This is in keeping with the findings from adult humans that reported no sex-dependent differences in the HA stem-specific antibody [28]. With that said, we only evaluated these antibodies in our model at d21 following boost. In our mother-reared cohort, we found that the amount of stem-specific antibodies continued to increase through the 3 months following vaccination [26]. Unfortunately, the number of animals present in that cohort is too low for the appropriate interrogation of sex-based effects. Thus, whether differences are present at later times awaits further exploration.

Sex-associated differences in PR8-specific IgG were only observed when R848 agonists were included in the vaccine. R848 binds to both TLR7 and TLR8 [33], while flagellin is a ligand for TLR5. These TLR molecules differ in cellular distribution and in cellular location [34]. TLR7 and TLR8 are present in endosomes, while TLR5 is on the cell surface (for review, see [34]). TLR7 is found on a variety of cells, including PDC, T cells, and B cells [35,36,37], while TLR8 is present on most monocytes, DC, and Tregs [35,38]. TLR5 is on DC, monocytes, and T cells [35,39]. Thus, while both adjuvants target T cells, DC, and monocytes, R848 can also promote naïve B cell activation.

The *TLR7* and *TLR8* genes are located on the X chromosome [17,18]. This is in contrast to the gene for *TLR5*, which resides in chromosome 1 [40]. Localization on the X chromosome accounts for the higher TLR7/8 expression reported in females afforded by an incomplete X inactivation [17,18]. Escape from X inactivation occurs in 30% of PDCs, monocytes, and B cells [17]. One outcome of higher TLR7 expression is the increased production of IFNα on a per-cell basis [41]. Type 1 IFN can enhance B cell responses induced as a result of BCR engagement [42]. Thus, increased IFN production resulting from TLR7 engagement may drive these responses. TLR7 engagement in B cells also promotes the enhanced proliferation of B cells that have the biallelic expression of the *TLR7* gene [17]. Enhanced B cell activation with the R848 adjuvant aligns with our previous analysis of draining lymph nodes at 24h following vaccination, where we found an increase in CD69^+^ B cells compared to lymph nodes from animals vaccinated with flagellin [43]. The inability of flagellin to promote increased antibodies in female newborns suggests that modulation in MyD88, IRAK-1, and IRAK-4 molecules involved in the initiation of signaling are not the basis for the improved response in females as both TLR7/8 and TLR5 pathways share these factors [44].

Previous studies carried out in adult mice using an inactivated influenza virus or a split virus vaccine recapitulate the female-associated improved response in both inbred and outbred strains [45,46]. The generalized stronger immune response reported in females is proposed to be the result of different gonadotropin, e.g., estradiol and testosterone, profiles in males versus females, each of which can regulate multiple cells of the immune system [7]. The absence of higher antibody levels in female newborns administered the inactivated influenza virus alone may indicate that the effect of hormonal regulation on immune cells have not yet manifested. We propose that the female-restricted effects of R848 through TLR7/8 binding is independent of the hormonal environment present following birth and is instead the result of increased TLR7/8 expression in females.

When influenza vaccine was administered to adult mice, increased antibodies were associated with lower splenic Tfr/Tfh and Tfr/GC ratios [46]. The reduced ratios were attributable to a greater absolute number of Tfh and GC B cells in female mice, suggesting a model wherein females support increased the recruitment, activation, and/or expansion of Tfh and GC B cells compared to males. R848 may be facilitating this through the engagement of TLR7 on B cells and T cells.

Interestingly, a sex-associated increase in the influenza-specific neutralizing antibody was observed only when both adjuvants were present. In comparing male newborns vaccinated with IPR8-R848 versus IPR8-R848+flg, no significant difference in the neutralizing antibody was detected. In contrast, there was a statistically significant increase in females vaccinated in the presence of both adjuvants versus R848 alone. Thus, the benefit of dual adjuvanted vaccine is only apparent in females. The generation of neutralizing antibodies is often associated with germinal center (GC) reactions. One possibility is that the high-level neutralizing antibody requires R848 engagement on newborn B cells, promoting their activation [43], together with flagellin, which has been reported to support germinal center formation [47].

The data reported here extend our understanding of the newborn immune system. Furthermore, they support the need to ensure that vaccine approaches are tested in both males and females. On a practical basis, vaccines must be designed to promote adequate responses in both sexes. As such, it is necessary to design approaches that are effective for the weaker responding group. This study has some limitations. The NHP model restricts the number of experimental conditions (e.g., vaccines, adjuvants) and time points that can be assessed. Further, here, we investigated inactivated influenza virus; thus, it is unclear whether our results are generalizable to all vaccines. This model opens the door to future studies to more fully understand the sex-dependent regulation of the immune system in newborns.

## 5. Conclusions

Here, we provide new insights garnered from the highly translational, pre-clinical NHP model. Firstly, sex-dependent differences to the whole inactivated influenza virus were not apparent in the absence of an adjuvant, suggesting that the full effects of the hormonal regulation of the immune system may not yet be manifest in these young infants. Secondly, R848, but not flagellin, could drive an increased response in females. Thus, not all adjuvants drive greater antibodies in females compared to males. These findings increase our understanding of newborn immunity and have implications for the rationale design of vaccines for use in this population.

## Data Availability

The data supporting this publication are available upon request.

## Supplementary Materials

The following supporting information can be downloaded at: https://www.mdpi.com/article/10.3390/vaccines12040415/s1, Figure S1: Anti-influenza IgG responses are similar in vaccinated nursery-reared versus mother-reared infants. Influenza virus-specific IgG antibody in plasma was measured by ELISA on d21 p.b.
