# Supplementary material for: Sex-Dependent Effects on Influenza-Specific Antibody Quantity and Neutralizing Activity following Vaccination of Newborn Non-Human Primates Is Determined by Adjuvants
Source: Vaccines (Basel). 2024 Apr 15;12(4):415. doi: 10.3390/vaccines12040415 (PMC11054256; doi:10.3390/vaccines12040415)
Supplement: Supplementary file 1 [file vaccines-2914378-supplementary.pdf › vaccines-2914378-supplementary.pdf]

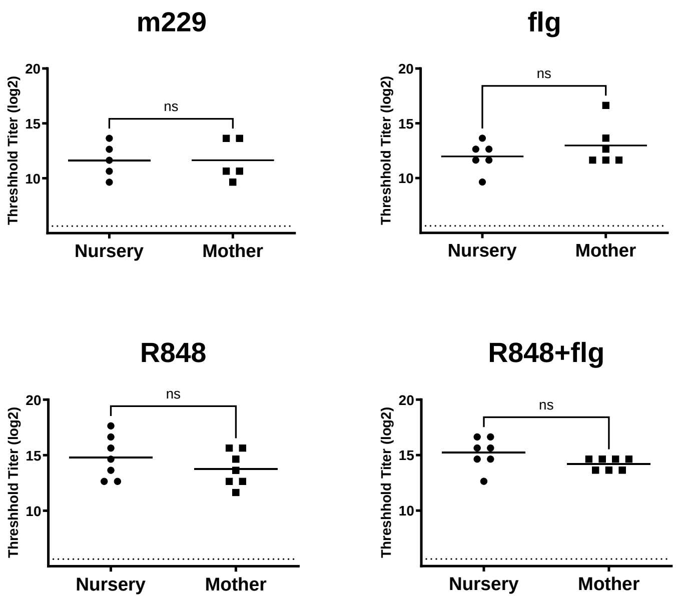

Supplementary Figure S1. Anti-influenza IgG responses are similar in vaccinated nursery-reared versus mother-reared infants. Influenza virus-specific IgG antibody in plasma was measured by ELISA on d21 p.b.
